# Development of Hypertolerant Strain of *Yarrowia lipolytica* Accumulating Succinic Acid Using High Levels of Acetate

**DOI:** 10.1021/acssuschemeng.2c02408

**Published:** 2022-08-09

**Authors:** Vivek Narisetty, Ashish A. Prabhu, Rajesh Reddy Bommareddy, Rylan Cox, Deepti Agrawal, Ashish Misra, M. Ali Haider, Amit Bhatnagar, Ashok Pandey, Vinod Kumar

**Affiliations:** † School of Water, Energy and Environment, Cranfield University, Cranfield MK43 0AL, United Kingdom; ‡ Department of Applied Sciences, Northumbria University, Newcastle Upon Tyne NE1 8ST, United Kingdom; § School of Aerospace, Transport and Manufacturing, Cranfield University, Wharley End MK43 0AL, United Kingdom; ∥ Biochemistry and Biotechnology Area, Material Resource Efficiency Division, CSIR- Indian Institute of Petroleum, Mohkampur, Dehradun 248005, India; ⊥ Department of Biochemical Engineering& Biotechnology, Indian Institute of Technology Delhi, New Delhi 110016, India; # Department of Chemical Engineering, Indian Institute of Technology Delhi, New Delhi 110016, India; ¶ Department of Separation Science, LUT School of Engineering Science, LUT University, Sammonkatu 12, Mikkeli FI-50130, Finland; ∇ Centre for Innovation and Translational Research, CSIR-Indian Institute of Toxicology Research, Lucknow 226 001, India; ○ Centre for Energy and Environmental Sustainability, Lucknow 226 029, India; ⧫ Sustainability Cluster, School of Engineering, University of Petroleum and Energy Studies, Dehradun 248 007, India

**Keywords:** acetate, succinic acid, Yarrowia lipolytica, acetyl-CoA synthase, adaptive laboratory evolution

## Abstract

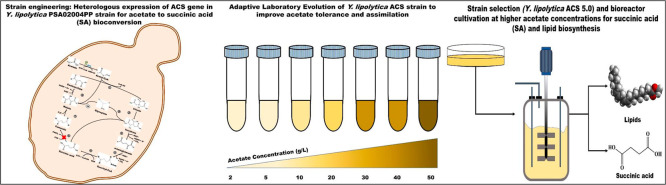

Acetate is emerging as a promising feedstock for biorefineries as it can serve as an alternate carbon source for microbial cell factories. In this study, we expressed acetyl-CoA synthase in *Yarrowia lipolytica* PSA02004PP, and the recombinant strain grew on acetate as the sole carbon source and accumulated succinic acid or succinate (SA). Unlike traditional feedstocks, acetate is a toxic substrate for microorganisms; therefore, the recombinant strain was further subjected to adaptive laboratory evolution to alleviate toxicity and improve tolerance against acetate. At high acetate concentrations, the adapted strain *Y. lipolytica* ACS 5.0 grew rapidly and accumulated lipids and SA. Bioreactor cultivation of ACS 5.0 with 22.5 g/L acetate in a batch mode resulted in a maximum cell OD_600_ of 9.2, with lipid and SA accumulation being 0.84 and 5.1 g/L, respectively. However, its fed-batch cultivation yielded a cell OD_600_ of 23.5, SA titer of 6.5 g/L, and lipid production of 1.5 g/L with an acetate uptake rate of 0.2 g/L h, about 2.86 times higher than the parent strain. Cofermentation of acetate and glucose significantly enhanced the SA titer and lipid accumulation to 12.2 and 1.8 g/L, respectively, with marginal increment in cell growth (OD_600_: 26.7). Furthermore, metabolic flux analysis has drawn insights into utilizing acetate for the production of metabolites that are downstream to acetyl-CoA. To the best of our knowledge, this is the first report on SA production from acetate by *Y. lipolytica* and demonstrates a path for direct valorization of sugar-rich biomass hydrolysates with elevated acetate levels to SA.

## Abstract 2

The ACS gene was expressed in *Yarrowia lipolytica* and evolved on acetate for succinate production. Stable hypertolerant *Y. lipolytica* was obtained after adapting on 5% acetate. *Y. lipolytica* ACS 5.0 attained robust growth (OD600: 23.5) when fed with acetate. ACS 5.0 produced 6.5 g/L succinate and 1.5 g/L lipid from acetate. Acetate-glucose cofermentation significantly improved the succinate titer by 87.7%. Adaptive evolution of acetyl-CoA synthase overexpressed *Y. lipolytica* for improved acetate tolerance and assimilation for succinic acid and lipid biosynthesis.

## 1 Introduction

The nonrenewable nature of fossil fuels, along with increased concerns about their depletion, nondegradable behavior, and environmental problems, such as greenhouse gas emissions and global climatic changes caused by their uses, has spurred the research for sustainable biomanufacturing.^1^ The first-generation biorefinery is very successful, but competing applications of these feedstocks in the food and feed industries have driven the search for alternative feedstocks. As a result, in recent decades, emphasis has been diverted toward using nonedible materials rich in fermentable carbon, most of which are from agroindustrial sectors.^2−4^ Acetic acid (CH_3_COOH) or acetate (CH_3_COO^–^), a C2 carboxylic acid, is a lucrative, noncompeting, and underexploited carbon source. It is emerging as a promising feedstock for biorefineries and industrial microbiology. Acetate can be manufactured through petrochemical and biotechnological routes using nonfood and feed competing resources and is cheaper than sugars. Conventionally, acetate is manufactured through the petrochemical route. However, with an upsurge in demand for biobased products, green acetate from biogenic processes, such as microbial fermentation and anaerobic digestion, has become a feedstock with significant interest. Besides, acetate is an imperative constituent of biomass hydrolysates and industrial wastewater streams where it is available in substantial amounts. Therefore, acetate and acetate-containing waste streams have drawn attention and become attractive as a low-cost and potential next-generation carbonaceous feedstock for the synthesis of chemicals, fuels, plastics, and so on via microbial routes.^5,6^

Succinic acid (SA), a C4 dicarboxylic acid, is a top platform chemical with broad applications in the chemical, pharmaceutical, and food industries.^4,7^ SA is an essential precursor for the production of 1,4-butanediol, tetrahydrofuran, and biodegradable polyesters such as polybutylene succinate. The traditional route of SA production involves either carbonylation of ethylene glycol, oxidation of 1,4-butanediol, or hydrogenation of maleic acid. However, due to the unsustainability and risks of chemical routes on the environment, biological processes involving renewable feedstocks have gained significant interest.^8,9^ Several bacterial and yeast strains have been employed for the bioproduction of SA from various carbon sources.^10−12^ The bacterial strains are very sensitive to pH fluctuations and require pH control between 6.0 and 8.0, yielding SA in salt rather than the acid form. The existence of SA in the salt form complicates downstream processing and makes the process expensive.^13^ On the other hand, yeasts with high tolerance to changes in pH and naturally predisposed to grow below pH 4.0 are the potential hosts to produce organic acids.^14^ The pK_*a*_ values of SA are 4.2 (pK_*a*_1) and 5.6 (pK_*a*_2), and approximately 80% of SA will exist in its protonated form at a pH < 4. This unionized form of SA facilitates its downstream processing from fermentation broth and improves the process economics as neutralization, and then acidification can be bypassed.^15^

Over the last few years, a nonconventional yeast, *Y. lipolytica*, is on the spotlight due to its versatile characteristics. The yeast strain has a well-annotated genomic model and metabolic tools for genetic manipulation. Metabolically, it assimilates a wide range of carbon sources, such as sugars, glycerol, and lipids, attains high cell density during growth and can withstand adverse conditions such as a low pH and high salt concentration. Furthermore, various products such as enzymes and lipids obtained by homologous or heterologous gene expression in this yeast have been conferred GRAS (generally regarded as safe) status by the US-FDA. The yeast has a very active carbon flux toward the TCA cycle, leading to the production of several intermediates such as citric acid, isocitric acid, α-ketoglutaric acid, SA, and so on.^16,17^ SA can be an intermediate of the oxidative or reductive TCA cycle. Owing to differential thermodynamics and other regulating factors, *Y. lipolytica* prefers the oxidative over the reductive pathway, utilizing different carbon sources such as glucose and glycerol.^13^ The amount of acid or base required for controlling pH during the SA production by *Y. lipolytica* can be lower compared to the bacterial SA producers as yeast can accumulate SA even at a low pH (<4.0).^13^*Y. lipolytica* can utilize a wide range of carbon sources, including glucose, glycerol, alkanes, and different classes of lipids.^16−18^ The yeast can also metabolize volatile fatty acids, including acetate, into lipids.^19^ Presently, only scarce information on SA production from acetate by *Y. lipolytica* is available.

Our previous work quantified acetate in higher titers than the main product (SA) during the SA biosynthesis.^13^ Traditionally, acetate is a microbial growth inhibitor and impairs their metabolic efficiency. The current work aimed to develop a *Y. lipolytica* strain as a cell factory for producing SA from acetate as a carbon source. The present study is a demonstration wherein acetate obtained as a byproduct during yeast metabolism was diverted toward SA. The work was started with heterologous expression of acetyl-CoA synthase in *Y. lipolytica* PSA02004PP; the recombinant strain developed from our previous work.^13^ The constructed strain was subjected to adaptive laboratory evolution (ALE) to alleviate toxicity and improve the tolerance against acetate, a toxic substrate for microorganisms. The evolved strain was cultured at different concentrations of acetate (10–50 g/L) as the sole carbon source under shake flask conditions and evaluated for cell growth and SA production, followed by cofermentation with acetate and glucose. After the shake flask, the process was validated in bioreactors under batch and fed-batch mode of cultivations with acetate and a mixture of acetate and glucose as carbon sources.

Furthermore, a small-scale compartmentalized metabolic network was used to analyze for the optimal SA production route from acetate using flux mode analysis. Metabolic fluxes were elucidated using the same model and experimental data. Insights were drawn into acetate metabolism to amplify SA production.

## 2 Materials and Methods

### 2.1 Chemicals and Materials

Q5 Taq DNA polymerase, restriction enzymes, and T4 DNA ligase were purchased from New England Biolab (Massachusetts, USA). The plasmid and DNA gel extraction kit were obtained from NBS Biologicals (Cambridgeshire, UK). *Escherichia coli* DH5α used for clone propagation and *E. coli* BL21 (DE3) were procured from Thermo Fischer Scientific (Massachusetts, USA). The plasmid JMP62 LeuTEF was a kind gift from Dr Rodrigo Ledesma-Amaro, Imperial College London, UK. All other chemicals used in this study were purchased from Sigma-Aldrich (Missouri, USA) and of analytical grade until otherwise stated.

### 2.2 Microorganism, Cultivation, and Maintenance

*Y. lipolytica* PSA02004PP, a recombinant strain previously developed in our laboratory,^13^ was used for acetyl-CoA synthase expression studies. The recombinant strain was preserved as glycerol (20% v/v) stocks at −80 °C and maintained on the Petri plates containing the YPX agar medium (1% yeast extract, 2% peptone, 2% xylose, and 1.8% agar) at pH 6.8 and 30 °C. The preculture was grown in 250 mL Erlenmeyer flasks with 50 mL of the YPX broth with an initial pH of 6.8. The sterile medium was inoculated by a loopful of a 24 h old culture grown on a YPX plate. Furthermore, the flask was incubated for 24 h at 30 °C on a rotary shaker at an agitation rate of 250 rpm.

### 2.3 Cloning and Expression of Acetyl-CoA Synthase (ACS) Gene in the *Y. lipolytica* Strain

The *E. coli* BL21 (DE3) genomic DNA was isolated following the protocol developed by He.^20^ The *acs* gene encoding for acetyl-CoA synthase was amplified from the isolated genomic DNA using the forward primer 5′GTAGGATCCATGAGCCAAATTCACAAACAC3′ flanked by *BamHI* and the reverse primer 5′ ATTCCTAGGTTACGATGGCATCGCGATAGC3′ flanked by *AvrII*. The PCR product of the *acs* gene (1.96 kb) was ligated into the BamHI/AvrII site of the JMP62 LeuTEF plasmid. The JMP62 LeuTEF plasmid is an integrative plasmid comprising a zeta sequence and mediates genome integration through a single crossover event. The modified plasmid was designated as JMP-*ACS*. JMP-*ACS* was linearised using *NotI,* which yields two fragments. The first fragment consists of the kanamycin resistance gene and the origin of replication for bacteria. The second fragment comprises the LEU2 marker and the expression cassette (Acetyl CoA synthase gene and TEF promoter) flanked by the zeta region. The latter fragment was purified in an agarose gel and transformed into the *Y. lipolytica* PSA02004PP strain using the lithium acetate method described earlier.^21^ The positive clones were selected on YNB Leu plates, and the recombinant strain was designated as *Y. lipolytica* PSA02004PP-ACS. Furthermore, positive transformants were confirmed by performing PCR using the genomic DNA of the *Y. lipolytica* PSA02004PP-ACS strain as the template.

### 2.4 ALE of the *Y. lipolytica* ACS Strain

The ALE was carried out to improve the resistance and robustness of the ACS strain at elevated acetate levels. The YPA medium containing (g/L)10, yeast extract; 20, peptone; and 2–50, sodium acetate, was used for this purpose. The YPA medium with the initial pH adjusted to 6.8 was used to domesticate the *Y. lipolytica* PSA02004PP-ACS strain in a flask culture at 30 °C with an agitation rate of 250 rpm. The ALE process was carried out in two stages. In the first stage, the recombinant strain was cultured sequentially in a liquid broth with an initial acetate concentration of 2, 10, 20, 30, 40, and 50 g/L. At each concentration, the strain was incubated for 48 h before transferring to the next higher concentration. Following the incubation at 50 g/L acetate, the evolved strain was subcultured thrice in the flasks containing 50 g/L acetate until identical OD_600_ values were obtained. The culture was then advanced to the second stage, where the adapted culture was spread on YPA agar plates with 50 g/L acetate. After the growth, 10 random colonies were selected and subcultured on YPA plates with 50 g/L acetate. Finally, the fast and well-grown colony was selected and reproduced in the liquid broth with acetate as the carbon source. The liquid culture was used to prepare glycerol stocks and working cell banks for further experiments to evaluate SA and lipid production.

### 2.5 Shake Flask Experiments

The submerged fermentation in shake flasks was conducted in 500 mL Erlenmeyer flasks containing 100 mL of the YPA medium with varying acetate concentrations (10–50 g/L). In the case of cofermentation, acetate was supplemented with 20 g/L glucose. The initial pH of the production media before inoculation was adjusted to 6.8 using 5M NaOH, and every 24 h, the pH was measured and adjusted to 6.8 under sterile conditions. As explained earlier, the freshly grown preinoculum in the YPX broth was used to inoculate the production media at an OD_600_ of 0.1 and kept for incubation at 30 °C with an agitation rate of 250 rpm.

### 2.6 Bioreactor Cultivation

Batch and fed-batch experiments were performed in a 3 L bench-top bioreactor (Electrolab Bioreactors, UK) made of borosilicate glass and all the metal work with 316 L stainless steel. The vessel is designed with a height: depth ratio of ∼2:1 at a maximum working volume of 2.5 L working volume. This study was carried out using the optimal concentration of acetate as the sole carbon source, obtained during the shake flask experiments and in cofermentation. The pH, temperature, agitation speed, and aeration (air from a compressor) rate were controlled throughout the cultivation at 6.8, 30 °C, 400 rpm, and 2.0 vvm, respectively. In fed-batch fermentation, the acetate concentration was maintained at or above 5 g/L with the concentrated feed containing 100 g/L acetate.

### 2.7 Metabolic Flux Analysis

The metabolic model of *Y. lipolytica* was constructed as previously described.^22,23^ Briefly, the compartmentalized metabolic model included acetyl-CoA synthase facilitating acetate uptake and conversion to acetyl-CoA. Central metabolic enzymatic reactions for the pentose phosphate pathway, gluconeogenesis, TCA cycle, glyoxylate shunt, and biomass constituents were considered. To further reduce the complexity of the pathway during flux estimation, only mitochondrial malic enzyme and mitochondrial NADP- dependent isocitrate dehydrogenase were considered. The truncated model was considered from the available information; for instance, only specific transport reactions between mitochondria and cytosol, viz. unidirectional pyruvate shuttle, acetyl-CoA, and oxaloacetate from the cytosol to mitochondria were considered, and the list of the metabolic reactions are listed in Table S1. Calculated extracellular fluxes, that is, the acetate uptake rate (set to 100%), SA, biomass, and lipid production rate, were used to constrain the model. Flux prediction was performed using the extracellular fluxes described previously,^24^ where estimations were based on the variance-weighted least square method and tested for consistency by the test function h at a confidence level of 0.90 and with a degree of freedom 1 using CellNetAnalyser^25^ in MATLAB 2021b. The stoichiometric model is comprised of 70 reactions and 61 metabolites (Table S1). Based on this model, an optimal SA production scenario was also estimated from acetate. For comparison purposes using elementary mode analysis, acetate uptake reactions were replaced with glucose or glycerol uptake reactions.

### 2.8 Lipid Quantification

Lipid quantification was only conducted on samples with the maximum OD_600_ reading. For this, 20 mL of the fermented broth was taken in a preweighed 50 mL centrifuge tube and centrifuged down at 2000 rpm for 10 min. The resulting pellet was resuspended in deionised H_2_O and washed twice to remove unwanted contaminants before the final pellet was frozen and freeze-dried until a constant weight was produced.

Lipid extraction was conducted with a minimum of 20 mg biomass using a modified Bligh and Dyer method.^26,27^ Initially, the known microbial biomass was suspended in 4 mL of methanol/chloroform solution (2:1 v/v) and ruptured using a sonication probe at 8 W for 5 min in an ice bath to ensure no oxidation of lipids during the process. Furthermore, 0.09% w/v NaCl solution was added immediately after sonication to aid the separation of solvent phases. The solution containing ruptured cells was centrifuged at 2000 g for 10 min. After the solvent phase separation, the bottom chloroform phase was pipetted into a preweighed glass vial. The above process, from the initial step of microbial biomass suspension in methanol/chloroform solution (2:1 v/v), was repeated twice until the chloroform layer pipetted out became clear to ensure that all lipids were extracted. The chloroform solution containing lipids was subjected to drying in a nitrogen evaporator maintained at 40 °C until a constant mass was recorded. Then, the lipid percentage per cell biomass was calculated using eq 1.
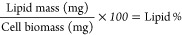
1

### 2.9 Analytical Techniques

Samples were withdrawn at regular intervals during the shake flask and bioreactor experiments to analyze cell growth (OD_600_), residual glucose, acetate, and SA concentrations. The cell growth was quantified by measuring the optical density at 600 nm in a 1 mm path length cuvette using a double beam spectrophotometer (Jenway 6310, UK). The substrate and metabolite concentrations were measured using a high-performance liquid chromatography (HPLC) system. The supernatant collected after the centrifugation at 10,000 rpm for 10 min to remove the microbial cells and other suspended solids was filtered through a 0.22 μm nylon membrane (Sartorius, Germany). The filtered samples with appropriate dilutions were loaded into the HPLC system equipped with a Rezex ROA-Organic Acid H + (Phenomenex, USA) column and connected with two detectors, refractive index and diode array detector, which measured sugars and organic acids, respectively. The mobile phase was 5.0 mM H_2_SO_4,_ and the flow rate was 0.4 and 0.6 mL/min for sugars and acids, respectively. All the experiments were carried out in triplicates, and the standard deviation never exceeded >10%.

## 3 Results

### 3.1 Construction of the Recombinant *Y. lipolytica* Strain Expressing Acetyl-CoA Synthase

**Figure 1:**
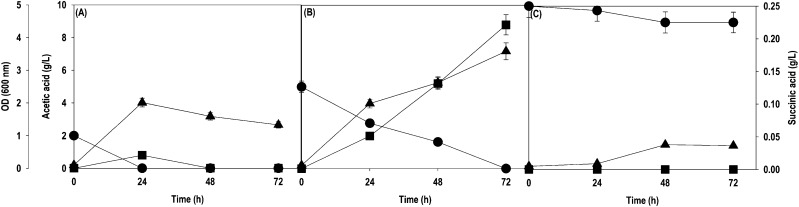
Time course profiles for acetate uptake, OD_600_, and SA production by the *Y. lipolytica* PSA02004PP-ACS strain during shake flask cultivation at different levels of acetate: (A) 2 g/L; (B) 5 g/L; (C) 10 g/L. Symbols: filled circle (acetate), filled triangle up (OD_600_), and filled square (SA).

Initially, the *acs* gene was amplified using the *E. coli* DNA as the template, and the 1.96 kb PCR product was ligated into the JMP62 LeuTEF plasmid at *BamHI* and *AvrII*restriction sites (Figure S1). The constructed JMP-ACS plasmid was transformed into the *Y. lipolytica* PSA02004PP strain. After the transformation, around 90 colonies were screened for growth on a medium containing acetate as the sole carbon source (YPA medium). The strain screening was carried out in 96-well plates with 150 μL of the YPA (containing 5 g/L acetate) medium inoculated with a preinoculum grown on the YPX medium for 18 h at 30 °C, at an OD_600_ of 0.1, and the culture was shaken constantly. The samples were withdrawn at constant intervals, and growth was monitored by measuring the optical density (OD_600_ nm) for 24 h. The recombinant strain showing the highest growth was investigated further and designated as *Y. lipolytica* PSA02004PP-ACS. The integration of the *acs* gene into the host genome was confirmed by PCR analysis using the primer set mentioned in Section 2.3. The strain was evaluated for cell growth and SA production on acetate as the sole carbon source. Figure 1 shows the time course profiles for acetate assimilation, cell growth (OD_600_), and SA production in shake flask experiments. When the recombinant strain was cultured on 2 g/L acetate, the carbon flux was mainly diverted toward cell growth. The acetate was completely utilized within 24 h leading to an OD_600_ value of 2.0 and a very low SA titer of 20 mg/L (Figure 1A). An increase in the initial acetate concentration to 5.0 g/L improved the OD_600_ and SA titers to 3.6 and 220 mg/L, respectively, concomitant with the complete depletion of acetate in 72 h (Figure 1B). Further increase in the acetate concentration to 10 g/L had a deleterious effect on cell growth and SA production. The recombinant strain assimilated only 1 g/L of supplied acetate in 48 h with an OD_600_ of 0.76, and no measurable level of SA was observed during the fermentation (Figure 1C). This study suggested that the recombinant strain required a strategy to perform at higher acetate concentrations in terms of robust growth and ability to accumulate high SA levels.

### 3.2 Enhancement in Acetate Tolerance of the *Y. lipolytica* ACS Strain via Adaptive Laboratory Evolution

**Figure 2:**
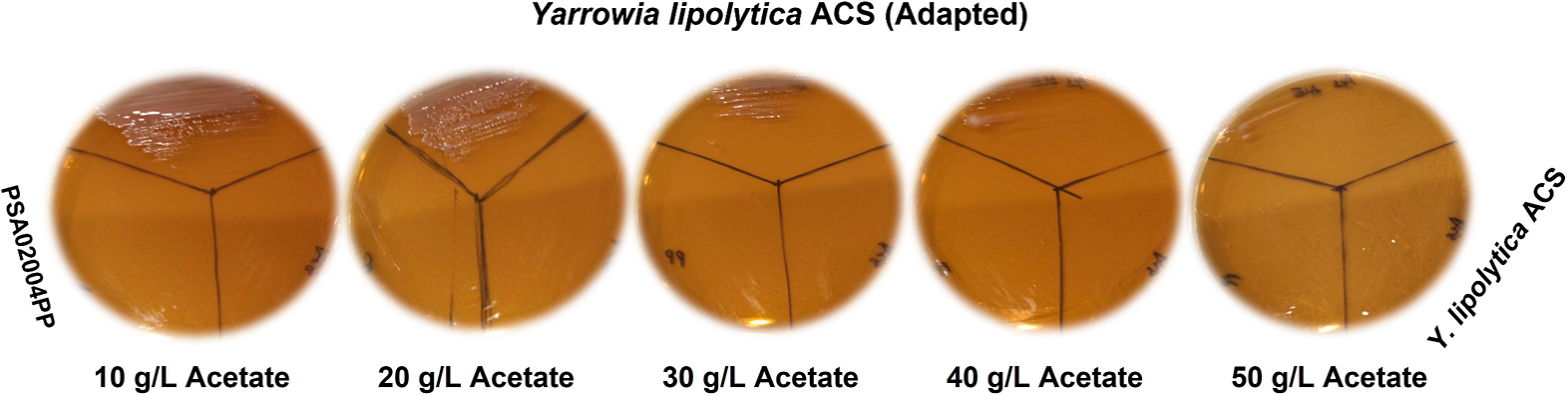
Cultivation of *Y. lipolytica* strains, PSA02004PP (modified to grow on xylose), PSA02004PP-ACS (modified to utilize acetate as C-source), and ACS 5.0 (adapted), on the YPA agar medium consisting of 10–50 g/L acetate as the sole carbon source.

SA biosynthesis using acetate as the carbon source can be through glyoxylate and the TCA cycle. The expression of glyoxylate shunt-related genes is vital for the growth and development of potent microbial hosts such as *Y. lipolytica* when cultivated on C2 substrates, such as acetate, to replenish TCA intermediates, such as SA or malate, and for the biosynthesis of precursors for gluconeogenesis and amino acids.^16,28^ The recombinant *Y. lipolytica* PSA02004PP-ACS strain grew on acetate as a carbon source at 2 and 5 g/L. However, deleterious impact on cell growth and SA production was noticed at 10 g/L acetate. To relieve the substrate toxicity, improve the strain tolerance, ameliorate the acetate assimilation efficiency, and attain a higher SA titer at elevated acetate levels, the strain was subjected to ALE. The strain was continuously subcultured on the YPA growth medium in a shake flask with a gradual increase in the acetate levels from 2 to 50 g/L. After attaining the steady OD_600_ of 12–15 in a shake flask at 50 g/L acetate, the culture was inoculated on a YPA agar plate with 50 g/L acetate. The colonies that appeared on the plate after 72 h were further subcultured on the YPA plates with same acetate levels. Only one colony appeared within 24 h, which was further subcultured and was denoted as ACS 5.0. The ACS 5.0 strain was further subcultured on the YPA plates with different concentrations of AA (10–50 g/L) along with two parent strains, *Y. lipolytica* PSA02004PP-ACS and *Y. lipolytica* PSA02004PP (Figure 2). Visualising yeast colonies on the agar plates showed that the adapted strain ACS 5.0 grew efficiently up to 30 g/L acetate, but it significantly reduced when cultured at 40 and 50 g/L acetate. On the contrary, both the parent strains did not show any growth at any of the employed acetate concentrations. To unveil the efficiency of the adapted *Y. lipolytica* ACS 5.0 strain, further investigations on cell growth, lipid, and SA biosynthesis were carried out in shake flask and bioreactor using acetate as the substrate/cosubstrate.

### 3.3 Shake Flask Cultivation of the Evolved *Y. lipolytica* ACS 5.0 Strain on Acetate as the Sole Carbon Source

**Figure 3:**
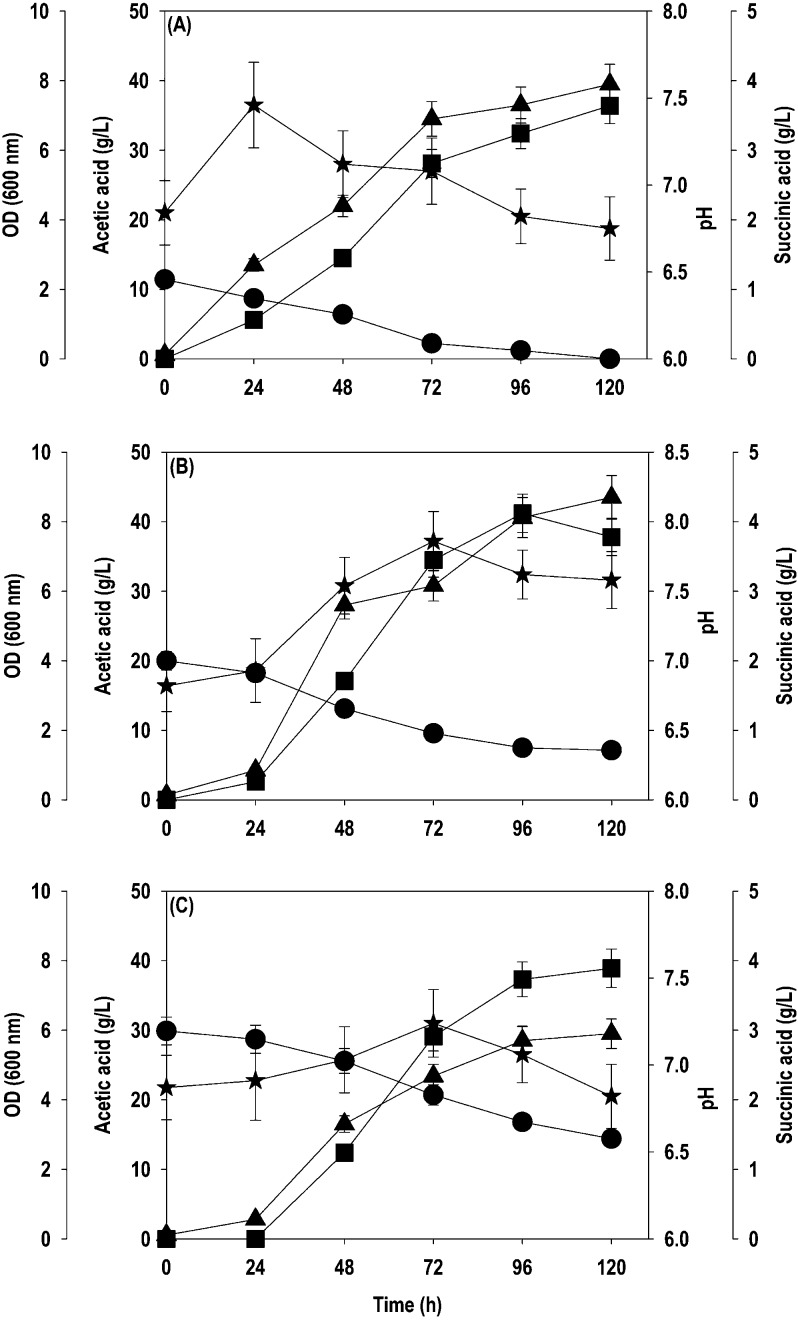
Time course profiles for acetate uptake, OD_600_, and SA production by the *Y. lipolytica*ACS 5.0 strain during shake flask cultivation at different levels of acetate: (A) 10 g/L; (B) 20 g/L; (C) 30 g/L. Symbols: filled circle (acetate), filled star (pH), filled triangle up (OD_600_), and filled square (SA).

The effect of initial acetate concentrations (10–50 g/L) on the assimilatory pattern, cell growth, SA biosynthesis, and change in pH by the adapted strain ACS 5.0 was evaluated in shake flask cultures under optimal culture conditions (30 °C, 250 rpm and initial pH 6.8). The time course profiles for acetate uptake, cell growth, SA formation, and pH are shown in Figure 3. At a 10 g/L initial acetate concentration, it was exhausted entirely within 120 h, resulting in cell OD_600_ and SA titer of 7.9 and 3.6 g/L, respectively (Figure 3A). However, 20 and 30 g/L acetate failed to be metabolized fully even after 120 h of cultivation. The cell OD_600_ and SA titer of 8.1 and 4.1 g/L, respectively, were achieved at 96 h with an initial acetate level of 20 g/L (Figure 3B). The residual acetate concentration of ∼7.5 g/L was observed at the end of fermentation, and the SA yield on acetate was 0.33 g/g. The situation aggravated at 30 g/L, where ∼52% of the supplied acetate was consumed with a cell OD_600_ of 5.9 and SA concentration of 3.9 g/L with a conversion yield of 0.25 g/g (Figure 3C). The accumulation of organic acids causes a reduction in pH, while their assimilation brings up the pH. The yeasts can resist the drop in pH as they are tolerant to acidic conditions, but an increased pH can negatively affect metabolism. We also found that acetate assimilation led to a gradual increase in pH that hindered the growth and development of the yeast. Hence, the pH was measured every 24 h and adjusted to 6.8 under sterile conditions to counter this. *Y. lipolytica* is an oleaginous yeast and is well known for intracellular accumulation of lipids.^16^ The lipids accumulated in the cell biomass were quantified at the end of fermentation, and a lipid concentration of 0.34, 0.61, and 0.41 g/L, representing 17.9, 24, and 44.1% of cell biomass, was obtained at initial acetate levels of 10, 20, and 30 g/L, respectively. Interestingly no other byproducts were observed during the cultivation on acetate, indicating that the acetate was consumed for cell growth, SA, and lipid accumulation. The performance of the evolved strain is highly encouraging as both the parent strains could not grow at all, even at 10 g/L acetate.

### 3.4 Cofermentation of Acetate and Glucose in a Shake Flask

**Figure 4:**
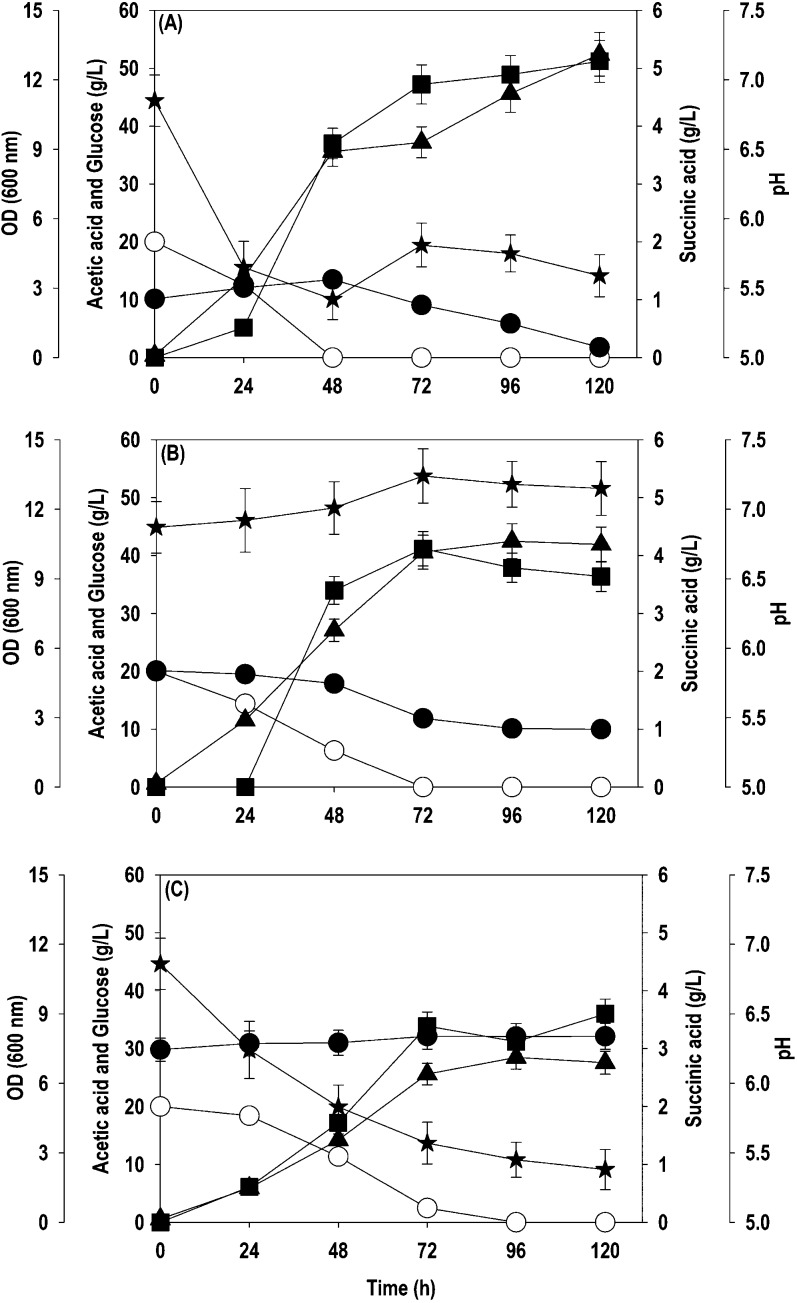
Glucose + acetate cofermentation by the *Y. lipolytica*ACS 5.0 strain in a shake flask at different levels of acetate: (A) 10 g/L; (B) 20 g/L; (C) 30 g/L. Symbols: empty circle (glucose), filled circle (acetate), filled star (pH), filled triangle up (OD_600_), and filled square (SA).

After culturing on acetate, cofermentation experiments were conducted with glucose (20 g/L) and acetate (10–50 g/L) as cosubstrates. It helped us to evaluate the impact of glucose addition on substrate assimilation, cell growth, and product formation. The idea of cofermentation was to accumulate a high biomass concentration on glucose, allowing rapid uptake of acetate and its subsequent conversion into SA. Glucose was the preferred carbon source, and acetate was majorly utilized after its depletion. The variations in glucose and acetate assimilation, OD_600_, SA formation, and pH during cofermentation are shown in Figure 4. During the cofermentation with 20 g/L glucose and 10 g/L acetate, glucose was completely metabolized within 48 h. It resulted in an OD_600_ of 8.9, SA titer of 3.7 g/L, and additional acetate production of 3.3 g/L, making a total acetate concentration of 13.5 g/L. The active assimilation of acetate commenced after 48 h, and 86.7% of it (11.7 g/L) was utilized, leading to further improvement in the OD_600_ and SA titer to 13.1 and 5.1 g/L, respectively (Figure 4A). The lag phase was extended at 20 g/L acetate, impeding the utilization of both glucose and acetate. At 72 h, the glucose was fully assimilated while 40% of the acetate was depleted, resulting in an OD_600_ of 10.2 and a SA titer of 4.1 g/L, with no further changes observed (Figure 4B). At 30 g/L acetate, 100% glucose utilization was observed, stretching the lag phase to 48 h with no acetate assimilation, resulting in a maximum cell density of 6.9 and SA titers of 3.6 g/L (Figure 4C). With 40 and 50 g/L acetate, the glucose consumption observed was only 51 and 15.1%, with a long lag phase and almost no uptake of acetate, leading to a cell OD_600_ and SA production of 5.6, 1.9, and 1.1, 0 g/L, respectively. Compared to fermentation with acetate as the sole carbon source, the lipid content of the cell biomass was higher during cofermentation. The amount of lipid accumulated at 10, 20, 30, and 40 g/L acetate was 0.87, 0.92, 1.04, and 0.96 g/L, which is 45.8, 44.3, 39.5, and 32.1% of the cell biomass generated.

### 3.5 Batch Cultivation of *Y. lipolytica* ACS 5.0 in a Bioreactor

**Figure 5:**
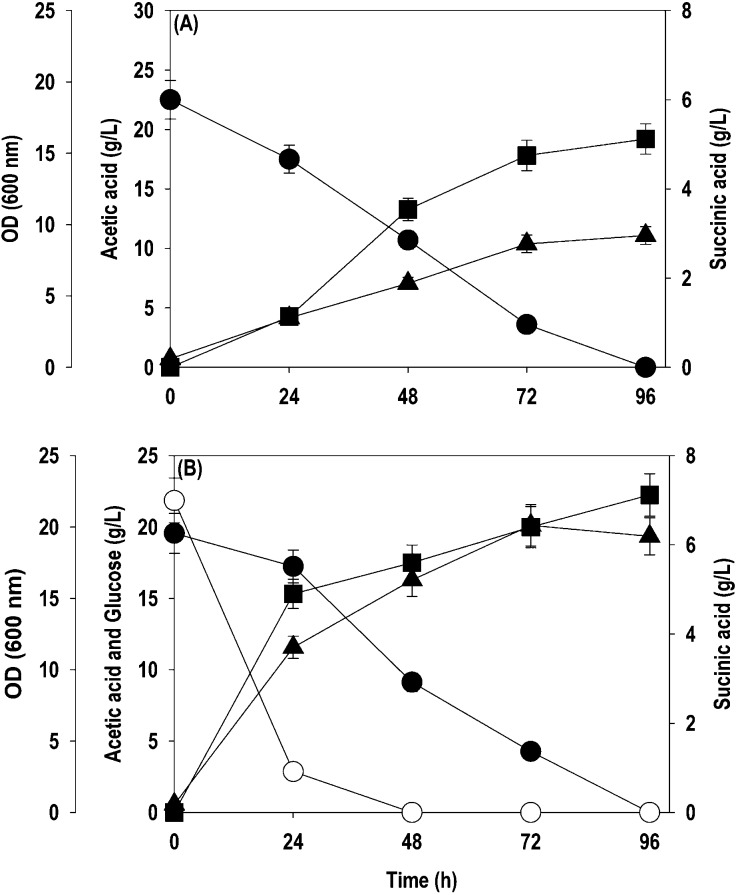
Batch cultivation of *Y. lipolytica*ACS 5.0 in a bioreactor: (A) acetate as the sole carbon source; (B) glucose + acetate cofermentation. Symbols: empty circle (glucose), filled circle (acetate), filled triangle up (OD_600_), and filled square (SA).

The batch cultivation of the recombinant strain ACS 5.0 using the optimal substrate concentrations was carried out in a bench-top bioreactor to validate and observe the strain efficiency. In shake flask experiments, approximately 15 g/L acetate was consumed within 72–96 h. It was envisaged that bioreactor cultivation under controlled conditions of pH and aeration would enhance acetate assimilation. Therefore, fermentation and cofermentation experiments in the bioreactor were started with an initial acetate level of 20–23 g/L. In the case of acetate as the sole carbon source, substrate uptake was faster in the bioreactor than in the shake flask, where ∼23 g/L acetate was exhausted. In contrast, at similar concentrations, a residual acetate level of 7.1 g/L was observed in the shake flask culture even after 120 h. The evolved ACS 5.0 strain attained a maximum cell growth (OD_600_) of 9.2, lipid concentration of 0.84 g/L corresponding to 45.8% of cell biomass, and SA titers of 5.1 g/L (Figure 5A), with a conversion yield of 0.23 g SA/g.

The cofermentation study in the bioreactor suggests that glucose (20 g/L) facilitated the uptake of acetate, and all the acetate was exhausted, while in the shake flask, ∼50% acetate was left unconsumed. Glucose depleted within 24–48 h, as observed earlier, following the maximum acetate assimilation. The supplementation of glucose as the cosubstrate caused a notable increment in cell growth, SA titers, and lipid accumulation. The highest cell OD_600_, SA titer, and lipid accumulation of 20.1, 7.1 g/L, and 1.24 g/L were achieved during cofermentation on glucose and acetate (Figure 5B). Furthermore, similar to the shake flask experiments, no byproducts were formed during single substrate and cosubstrate fermentation.

### 3.6 Fed-Batch Cultivation of *Y. lipolytica* ACS 5.0 in a Bioreactor

**Figure 6:**
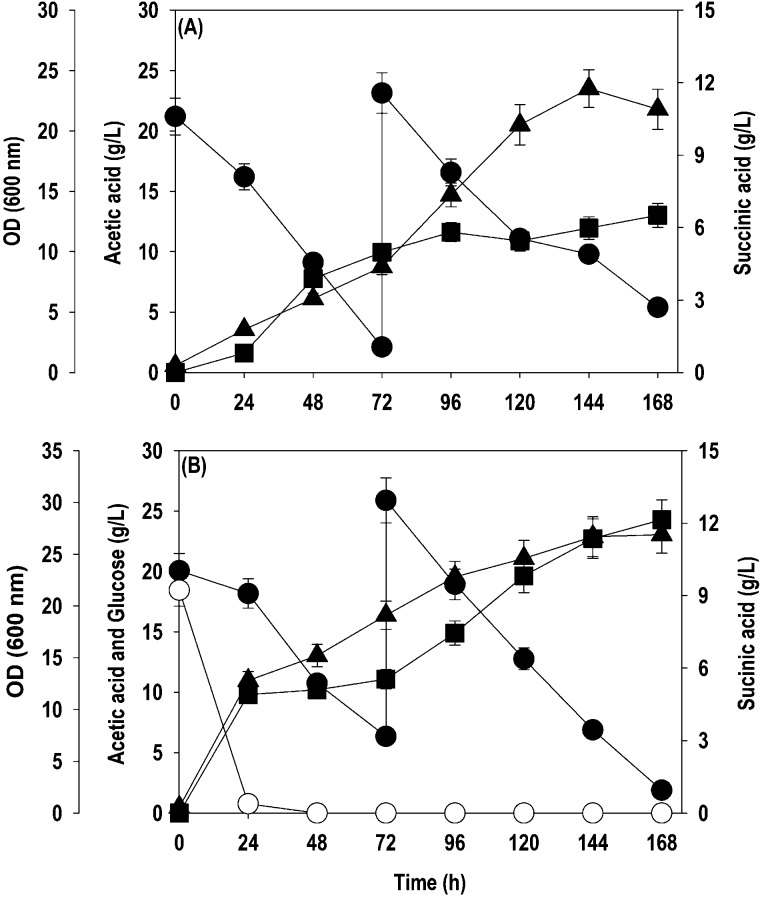
Kinetics of acetate uptake, OD_600_, and SA production by *Y. lipolytica* ACS 5.0 during the fed-batch cultivation in a bioreactor: (A) acetate as the sole carbon source; (B) glucose + acetate cofermentation. Symbols: empty circle (glucose), filled circle (acetate), filled triangle up (OD_600_), and filled square (SA).

During the shake flask and bioreactor experiments in the batch mode, we found that an acetate concentration >20 g/L is inhibitory for cell growth and SA biosynthesis. Fed-batch cultivation is preferred to circumvent the said issue, where the limiting substrate is added into the bioreactor in a controlled fashion, eliminating the substrate-mediated inhibition and increasing the end-product titers. Like the batch fermentation, two fed-batch fermentations were performed, one using acetate (20 g/L) as the sole carbon source and the other with a mixture of acetate and glucose fed at 20 g/L each. A concentrated (100 g/L) CH_3_COONa solution was used for replenishing the acetate in the culture medium when its concentration dropped <5 g/L. Figure 6 shows the time course profiles of substrate (AA and glucose) consumption, cell growth, lipid, and SA accumulation during the fed-batch mode of cultivation. During single-substrate fed-batch cultivation, ∼90% of initially supplied acetate was assimilated in 72 h. Only ∼50% acetate assimilation occurred in the next 48 h when the culture was fed with another 20 g/L, followed by a slow uptake, and <6.0 g/L was metabolized between 120 and 168 h. The cell growth and SA production were concomitant with acetate assimilation. The cell growth and lipid production increased continuously from the beginning, and a maximum cell OD_600_ of 23.5 and lipid concentration of 1.5 g/L were obtained at 144 h. The SA production was rapid in the initial 72 h, where 5.0 g/L SA was accumulated, followed by a phase of slow productivity with the maximum titer being 6.5 g/L, peaking at 168 h (Figure 6A). During cofermentation, ∼95% of initial glucose depleted within 24 h, while active acetate consumption commenced after 24 h, with 68.3% being metabolized at 72 h. The first feeding of acetate (20 g/L) into the system began at 72 h, and it was entirely assimilated within 72–144 h, unlike single-substrate fed-batch fermentation. Glucose addition boosted the cell growth and reduced the lag phase during cofermentation, with OD_600_ reaching 15.2 within 48 h and peaking at 26.7 in 144 h. The lipid accumulation was higher during cofermentation in comparison to cultivation on acetate as the sole carbon source, reaching a maximum of 1.8 g/L in 144 h. SA biosynthesis also followed the same pattern, and 4.9 g/L SA was accumulated in 24 h, followed by a slow and steady increment in SA production, leading to a final SA titer of 12.2 g/L (Figure 6B).

### 3.7 Flux Analysis and the Optimal Succinate Production Route

**Figure 7:**
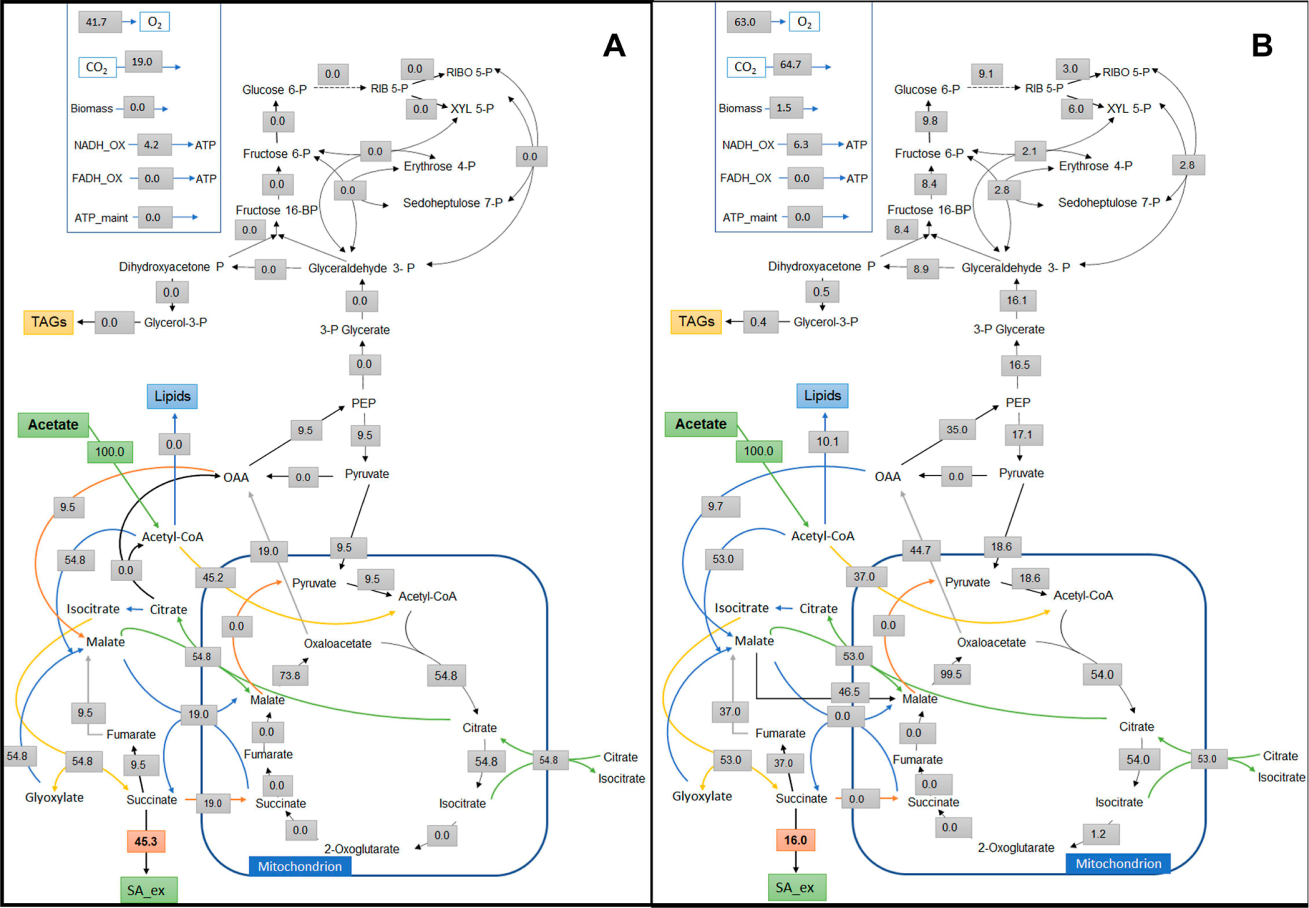
(A) Optimal SA production pathway fluxes from acetate in *Y. lipolytica*; (B) flux distribution from the experimental data. All values are in molar percentages of the acetate uptake rate, which was set to 100%.

The extracellular fluxes from the acetate bioreactor batch cultivation were used as constraints. The acetate uptake rate was fixed to an arbitrary value of 100. Other extracellular fluxes were determined from the yields of SA, lipogenic acetyl-CoA, glycerol-3-phosphate, and NADPH per 100 mol of acetate (Table S2). Lipogenic acetyl-CoA, glycerol-3-phosphate, and NADPH were calculated as previously described.^23^ For an optimal SA production from acetate, high flux values through the glyoxylate shunt are observed (Figure 7), a general phenomenon with substrates such as acetate or other organic acids entering the lower glycolytic pathway. In this scenario, carbon is directed chiefly toward the TCA and glyoxylate cycle. ATP required for acetate uptake is mainly generated via oxidative phosphorylation using NADH as the electron donor. Using this metabolic network, a theoretical maximum yield of 0.45 mol SA per mol of acetate was observed. As shown in Figure 7A, for the optimal production of SA from acetate, lower gluconeogenic fluxes and higher glyoxylate cycle fluxes are desirable. The best fit flux was estimated using the experimental data as depicted in Figure 7B. By comparing the optimal scenario with the present scenario, it was found that SA reached a theoretical maximum of 35% in the latter case. A certain portion of the cytosolic acetyl-CoA pool was used for lipid production. Higher gluconeogenic fluxes, as observed in this strain, require higher NADH and ATP, which, in turn, demands a higher oxygen requirement in the present scenario compared to the optimal SA production scenario. In this SA dehydrogenase mutant strain, the malate transporter plays a significant role in diverting the flux into the TCA cycle, where the malate dehydrogenase compensates for the required NADH. The pentose phosphate pathway mainly supplies NADPH for biomass and lipid production.

## 4 Discussion

Acetate is emerging as an alternative feedstock for biorefineries and is cheaper ($300–450/ton) than conventional substrates, such as glucose ($500/ton), with an annual global production of 12.9 million metric tons. Acetate can be synthesized via chemical and biological routes. The chemical method involves methanol carbonylation, ethylene oxidation, alkane oxidation, or during the acid pretreatment of lignocellulosic biomass. Acetate production through the biological route either involves fermentation of sugars and glycerol^5,6^ or acetogenic bacteria, which use C1 gases, such as CO and CO_2_, via the autotrophic Wood–Ljungdahl pathway, a promising way of biological carbon capture from the atmosphere and its fixation to acetyl CoA under anaerobic conditions, thereby reducing greenhouse gases.^29−31^

Thus, acetate can empower the development of cost-effective and sustainable bioprocesses without interfering with the food chain and conflicting with the usage of arable land. Moreover, acetate is one of the important and inevitable constituents of sugar-rich hydrolysates and prehydrolysates derived from lignocellulosic biomass. However, being toxic, acetate critically restrains the metabolic performance of microbes toward an efficient sugar uptake and subsequent valorization. In this scenario, before exploiting the sugar platform through the fermentative microbial route, either lignocellulosic hydrolysates should be detoxified by acetate removal, facilitating better biotransformation, or strain engineering should be adopted to consume acetate and further enhances robustness/tolerance for acetate.

In recent times, the use of acetate as a feedstock for microbial growth and production of biochemicals has gained interest. The acetate metabolism starts with its conversion to acetyl-CoA, an activated form of acetate and a key central metabolite, and acetate can be of potential significance if the desired end product can be generated from acetyl-CoA. Acetate is converted into acetyl-CoA by the action of acetate kinase-phosphotransacetylase (ACKA-PTA) and/or acetyl-CoA synthase (ACS). ACS functions anabolically and has a higher affinity for acetate, scavenging acetate at low concentrations. Acetate to acetyl-CoA conversion is a two-step process that begins with the formation of an acetyl-AMP enzyme complex and PPi from acetate and ATP, followed by a reaction with CoA-SH to produce acetyl-CoA and AMP. Acetyl-CoA is transformed into higher carbon compounds via the glyoxalate pathway and gluconeogenesis. The glyoxylate cycle is a modification of the TCA cycle where the metabolic requirements of the cell are met by using two-carbon compounds, such as acetate, in the absence of simple sugars.

**Figure 8:**
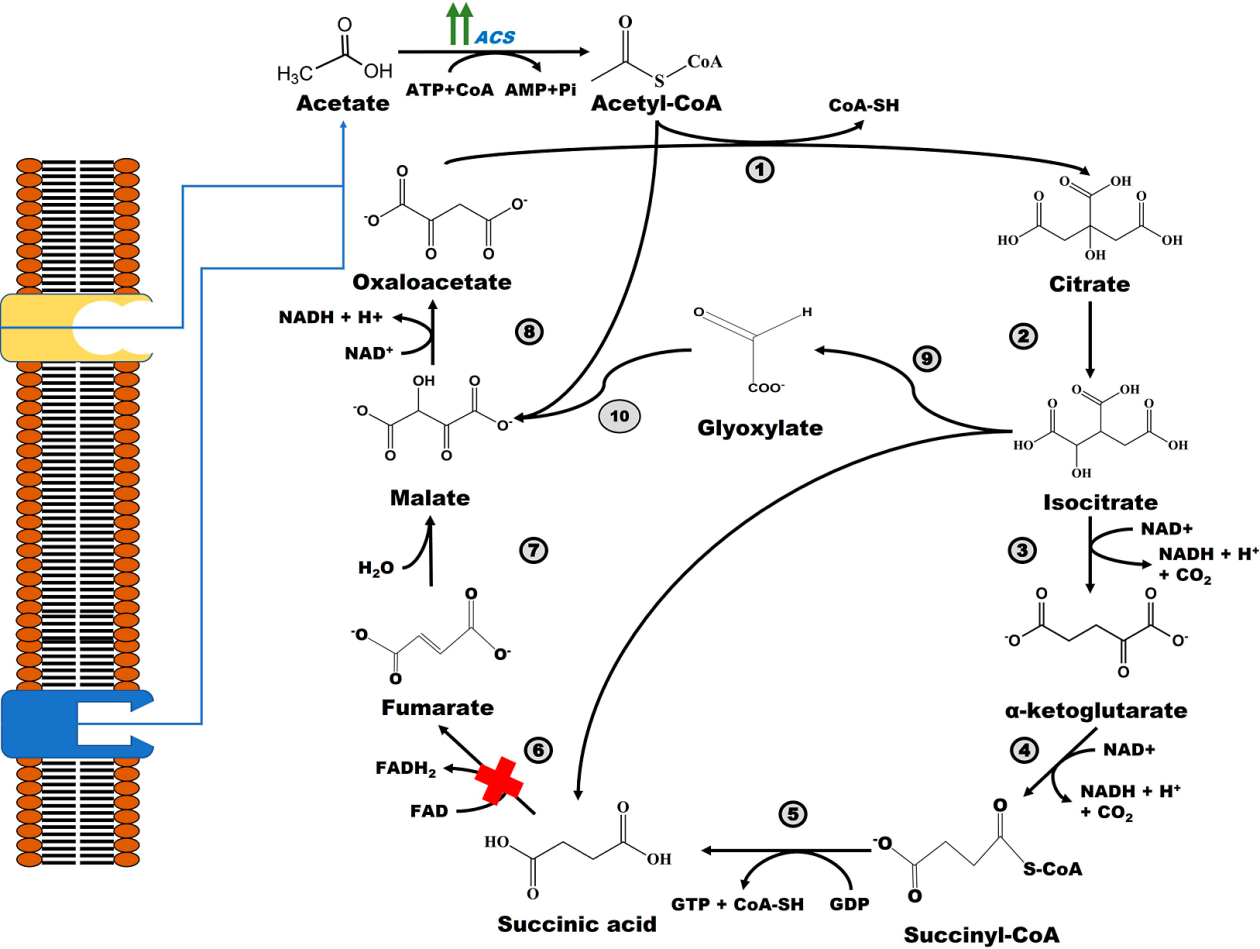
Metabolic pathway for SA production from acetate as the sole carbon source. Abbreviations: ACS: Acetyl-CoA synthase; 1: citrate synthase; 2: aconitase; 3: isocitrate dehydrogenase; 4: α-ketoglutarate dehydrogenase; 5: succinyl-CoA synthetase; 6: succinate dehydrogenase; 7: fumarase; 8: malate dehydrogenase; 9: isocitrate lyase; 10: malate synthase.

It is a shunt in which two decarboxylation steps of TCA are bypassed. Initially, acetate in its activated form (acetyl-CoA) is converted to citrate, which is later isomerized to isocitrate. Furthermore, isocitrate lyase catalyzes the splitting of isocitrate to SA and glyoxalate. The latter reacts with another acetyl-CoA molecule to generate malate, which is oxidized to oxaloacetate (Figure 8). One round of the cycle results in a net production of one molecule of SA with the following overall reaction (eq 2).
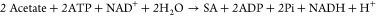
2

The maximum theoretical yield of SA on acetate is 0.5 mol/mol or 0.98 g/g. Moreover, being an oxidized chemical, the biosynthesis of SA requires low energy inputs; therefore, acetate can be a potential precursor for SA. Despite few studies on heterologous or homologous overexpression of ACS for lipid biosynthesis, no attempt has been made to couple acetate assimilation and SA accumulation in *Y. lipolytica*, to the best of our knowledge. In our previous work, we engineered *Y. lipolytica* for SA production from xylose, where acetate interestingly emerged as a major byproduct.^13^ In fact, the amount of acetate (25.0 g/L) accumulated was more than the desired product SA (22.3 g/L).^13^ Owing to the substantial acetate accumulation, the carbon flux toward SA was comparatively lower, resulting in low SA titers. Acetate is the most common fermentation inhibitor available in lignocellulosic hydrolysates, produced due to hydration of acetyl groups in the hemicellulosic fraction, which results in a more prolonged lag phase and decreased productivity. These factors motivated us to design a strain that could grow at high acetate levels and metabolize it to attain higher SA titers/yields. To this end, ACS from *E. coli* BL21 was expressed in *Y. lipolytica* PSA02004PP, a strain designed to accumulate SA from glucose and xylose via the oxidative TCA cycle in our previous work,^13^ and the resulting strain was designated as *Y. lipolytica* PSA02004PP-ACS. The chromosomal integration of a single copy of ACS conferred the recombinant strain to utilize acetate as the sole carbon source. When the *Y. lipolytica* PSA02004PP-ACS was fed with 2 and 5 g/L acetate, the strain displayed 100% substrate utilization with substantial cell growth and yielded 20 and 220 mg/L SA, respectively (Figure 1). However, the recombinant strain was not robust enough to grow at 10 g/L acetate, and a possible reason could be the toxic nature of acetate.

Along with the metabolic engineering strategies, ALE is a useful strategy for obtaining an efficient genotype or phenotype and optimizing the microbial chassis strains for an improved performance. Even the parent strain *Y. lipolytica* PSA02004 used in this study for ACS expression was found to have an impaired glucose assimilation when the *Ylsdh5* (Succinate dehydrogenase gene) was deleted for SA accumulation, but ALE on a glucose-based medium for 21 days restored the ability to use glucose.^32^ Therefore, we leveraged on the most promising tool, namely evolutionary engineering, to circumvent this critical problem. The recombinant strain expressing acetyl-CoA synthase was subjected to ALE in two stages. After several rounds of subculturing on the different acetate concentrations (10–50 g/L), the evolved strain (ACS 5.0) grew efficiently up to 30 g/L acetate on the Petri plate. It later displayed a significant decrease in the growth at 40 and 50 g/L (Figure 2). Shake flask cultivation showed full utilization of acetate when fed at 10 g/L, whereas at 20 and 30 g/L, the substrate was partially consumed with an OD_600_ and SA titer in the range of 5.9–8.1 and 3.6–4.1 g/L, respectively (Figure 3). Beyond these concentrations (40 and 50 g/L), severe inhibitions were observed, and the culture could not grow. Earlier, Seong and associates subjected an *E. coli* DSM01 strain for ALE in an M9 medium containing 5 g/L acetate for nine generations to efficiently utilize acetate.^33^ The evolved strain SBA01 was grown at different acetate concentrations ranging from 0.6 to 15 g/L. Similar to our results, the control strain could not grow under all the tested concentrations. On the other hand, SBA01 grew well, and the highest cell growth (OD_600_ ∼ 2.2) was obtained at 3.0 g/L acetate; thereafter, a continuous drop was noticed with very little growth at 15 g/L. The whole-genome sequencing revealed a mutation in *cspC* and *patZ,* conferring a competitive advantage to strain for growth on acetate through increment in ACS activity. Furthermore, the genes responsible for acetate utilization, the gluconeogenesis pathway, and the glyoxylate shunt were highly upregulated. The ACS pathway is expensive as it requires two ATP to convert acetate to acetyl-CoA, and to overcome this limitation, the SBA01 had increased expression of genes involved in the biosynthesis of ATP and NADH, which resulted in high levels of intracellular ATP.^33^ We strongly believe that similar to Seong et al. (2020), superior results obtained in the current work are contributed through a series of beneficial mutations and elevated gene expression of relevant pathways.

Process scale-up under the batch mode from the shake flask to the bioreactor led to complete utilization of acetate, both during single-substrate and cosubstrate fermentation, with the cell OD_600_ and SA titer being 9.2, 20.1 and 5.1, 7.1 g/L, respectively, as the physicochemical conditions were controlled (Figure 5). The SA and lipid accumulation on acetate further improved when cultivation was shifted from the batch to fed-batch mode. The SA and lipid titer enhanced from 5.1 and 0.84 g/L during the batch culture to 6.5 and 1.5 g/L, respectively, with fed-batch cultivation (Figure 6). The SA yield on acetate was reduced from the batch to fed-batch culture; however, OD_600_ was significantly increased, indicating more diversion of acetate carbon toward cell growth.

There are few reports where acetate has been used for lipid accumulation by *Y. lipolytica,*(16,34,35) but we did not come across any prior report on SA production from acetate by the yeast. Xu et al. developed an optimized semicontinuous system for the biological conversion of acetate to triacylglycerols (TAG).^36^*Y. lipolytica* MTYL065 overexpressing the acetyl-CoA carboxylase (*ACC1*) and diacylglycerol acyltransferase (*DGA1*) enzymes was employed for this purpose. The process used low-strength acetic acid in both the salt and acid form, while cross filtration modules were fitted to a bioreactor for cell recycling, and feeding of the substrate and nitrogen source was controlled in a way to reduce diversion of acetate to citrate and simultaneously maximize lipid accumulation, respectively. The high-density culture of the recombinant strain accumulated 115 g/L lipids with conversion yield and productivity of 0.16 g/g acetate and 0.8 g/L. h.^36^ In a similar approach, Chen et al. genetically modified the *Y. lipolytica* PO1f strain by overexpressing acetyl-CoA synthetase, acetyl-CoA carboxylase, and fatty acid synthase gene. The engineered strain amassed 25.7% lipids on acetate, which improved to 41.7% during glycerol + acetate cofermentation.^35^ In another approach, Hu et al. developed a two-stage integrated bioprocess, where in the first stage, the syngas coming from gasification of coal/natural gas/biomass was converted into acetic acid (AA) by *Moorella thermoacetica*, an acetogen with a high autotrophic flux to acetyl-CoA using the Wood–Ljungdahl pathway.^29^ The obtained AA was converted aerobically into microbial lipids by the engineered *Y. lipolytica* strain. The AA was not allowed to accumulate beyond 25 g/L to avoid its toxic effects. The accumulated AA was fed to a second bioreactor, and *Y. lipolytica* produced 18 g/L lipids with a lipid content of 36% using this acetate. The lipid titer and content improved to 46 g/L and 59%, respectively, when *Y. lipolytica* was separately cultured on acetate (3% v/v) with cell recycling.^29^

The SA production from acetate was attempted in *E. coli* MG1655 by Li et al., where they employed metabolic engineering approaches simultaneously to disrupt the TCA cycle, activate the glyoxalate cycle, and divert the carbon flux toward SA by enhancing the availability of common intermediates of TCA and glyoxalate cycle.^28^ As opposed to our results, the overexpression of acetyl-CoA synthase significantly inhibited the growth. However, the overexpression of citrate synthase in the triple mutant (Δ*sdhAB,* Δ*iclR*, Δ*maeB*) improved the SA titer from 6.86 to 16.45 mM (1.94 g/L) in 72 h with an SA yield of 0.91 g/g. Furthermore, a two-stage bioprocess was performed using the same strain, where cells were initially cultivated on the complex medium using glucose as the carbon source. After attaining the maximum OD_600_, citrate synthase was induced by addition of IPTG, and the cells were transferred to a minimal medium with CH_3_COONa as the carbon source and no nitrogen source. The cells accumulated 61.71 mM (7.3 g/L) of SA; however, the yield was reduced to 0.59 g/g.^28^ In their next study, the strain was further metabolically engineered to divert more carbon flux toward SA.^37^ The report involved multiple gene deletions and overexpression, which facilitated acetate utilization and maintained the NADH supply under aerobic conditions through an exogenous supply of formate. The recombinant strain (Δ*sdhAB,* Δ*iclR*, Δ*maeB* Δ*pckA_ackA-pta_gltA_fdh*) with exogenous addition of formate (at 10 mM) resulted in 30.9 mM (3.65 g/L) SA with a yield of 1.0 g/g within 72 h. The culture medium was supplemented with formate to provide more NADH (eq 3).^37^
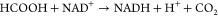
3

Considering these examples of *E. coli*, overexpression of merely one gene (ACS) in the present study together with ALE fetched more promising results by producing 6.5 g/L SA under the fed-batch mode of cultivation in a bioreactor. The result obtained in the current study is better than the previous literature reports and demonstrates *Y. lipolytica* ACS 5.0 as a promising strain for SA production from acetate.

Unlike traditional carbohydrates, acetate is a toxic substrate and cannot be used at high concentrations. In the present study, we anticipated that glucose fortification would stimulate high cell growth, enabling rapid assimilation of acetate and eventually higher SA production. Initially, cofermentation was performed with 20 g/L glucose and different acetate levels (10–50 g/L) in a flask culture. The presence of glucose improved cell growth and SA production, and all the acetate was metabolized at 10 g/L. However, glucose suppressed the utilization of acetate at higher levels (20 and 30 g/L), and as a result, a large amount of acetate was left unconsumed (Figure 4). The cofermentation in the bioreactor further improved the cell growth and SA titer. The batch culture yielded a cell OD_600_ and SA titer of 20.1 and 7.1 g/L, respectively. During the fed-batch culture, a significant increment in cell growth (OD_600_: 26.9) and SA titer (12.2 g/L) was achieved; however, a substantial amount of acetate was left unconsumed even at the end of fermentation. The cofermentation was carried out to achieve high SA titers by partially diverting glucose toward active cell generation so that high cell density may be exploited for the maximum bioconversion of acetate to SA. Though the said intent was partially fulfilled, it paved the way toward better bioconversion of lignocellulose-derived sugars in the presence of high acetate levels to SA. Similar to our work, Fontanille et al. used a two-stage fermentation, where *Y. lipolytica* was cultured on glucose/glycerol in stage one to achieve a high cell density culture, followed by feeding with acetate, which led to oil-rich biomass with a higher lipid content (15.7 g/L).^19^

From the flux analysis, we have elucidated the optimal route for SA production and flux distribution of *Y. lipolytica* ACS 5.0 using acetate as the sole carbon source (Figure 7A,B). It was observed from previous studies that under lipogenic conditions, NADPH for lipid production is mainly supplied by the PP pathway in *Y. lipolytica* when acetate was used as the sole carbon source.^16^ It is also evident in the current scenario that the PP pathway mainly supplies NADPH. For acetate assimilation, the glyoxylate cycle and gluconeogenic fluxes are crucial. These gluconeogenic fluxes required for biomass component production drive higher NADH and ATP requirements. Aiming to enhance SA production from acetate, one obvious target is reduced lipogenesis, which will enable a higher acetyl-CoA supply for SA production. It will further reduce the fluxes through the PP pathway and gluconeogenesis, reducing the carbon loss in the form of CO_2_ and lower ATP demands. Using elementary flux mode analysis, we compared the solution space for optimal SA production in *Y. lipolytica* on other substrates such as glucose and glycerol (Figures S2–S4). When acetate is used as a carbon source, there is a limited solution space or scope to balance biomass accumulation and SA production. The SA yield on acetate comes at the expense of biomass. With acetate as a carbon source, there have not been significant flux spaces, as observed when glucose or glycerol was used as the substrate. In a scenario where an SA yield of 0.26 mol/mol acetate with a biomass yield of 0.11 was estimated, the PP pathway reactions were predicted to be inactive. Mitochondrial isocitrate dehydrogenase primarily fulfils the anabolic NADPH demand in this scenario. Although the solution spaces are limited with acetate, when SA production is compared at half of the maximum theoretical biomass yields, SA yields of about 0.50 c mol/c mol acetate can be achieved. These yields are similar to when glucose (0.65 c mol/c mol) or glycerol (0.56 c mol/c mol) were used as substrates. In the current scenario where the SDH is inactive, a significant flux through the malate transporter is observed to transport malate from the cytosol to mitochondria, enabling a higher flux through mitochondrial malate dehydrogenase mainly for NADH generation. As observed here and in a previous study,^16^ there needs to be a fine-tuning of flux distribution between gluconeogenesis, the glyoxylate cycle, and TCA cycle for an efficient redox balance and energy requirement when acetate is used as a carbon source. As observed from the flux analysis (Figure 7A,B), the activities of PEP carboxykinase, pyruvate kinase, malate dehydrogenase, and the malate transporter are keys to achieving this balance.

## 5 Conclusions

Acetate is an inexpensive carbon substrate, which can be obtained readily and in high quantities from various agroindustrial waste streams via chemical and biochemical routes. The present study explores the possibility of using acetate as a feedstock for SA production by *Y. lipolytica*. The oleaginous yeast has been well investigated for SA production using glucose and glycerol. The *Y. lipolytica* ACS 5.0 strain developed by a combinatorial approach of genetic and evolutionary engineering showed no significant inhibition even at 20–50 g/L acetate when cultivated on solid agar or during submerged cultivation. The strain looks promising, where acetate (as the sole substrate) produced 6.5 g/L SA and 1.5 g/L lipids and displayed an OD_600_ of 23.5 during fed-batch cultivation. Furthermore, cosubstrate fermentation with glucose resulted in 12.2 g/L SA and 1.8 g/L lipids and an OD_600_ of 26.7. Though the SA titer achieved is far from the industrial scale, the results are promising from a carbon source well known for its toxicity and being considered a fermentation inhibitor. Our results strongly indicate that acetate is no longer a foe and can become a friend for biobased industries. Future work should be directed toward fine-tuning and balancing fluxes between gluconeogenesis and the glyoxylate cycle to divert more acetate toward SA and process optimization to improve TYP (titer, yield, and productivity) metrics.

## Availability of Data and Materials

All data generated or analyzed during this study are included in the manuscript.

## Supporting Information Available

Plasmid map of JMP62 LeuTEF, SA production performance by Y. lipolytica on acetate, glucose, and glycerol, HPLC chromatogram representing the consumption of acetate and bioproduction of SA, metabolic network of Y. lipolytica growing on acetate, glucose, and glycerol, elementary mode and flux analysis, molar values of TCA cycle intermediates extracellular flux in Y. lipolytica grown on acetate, and references (PDF)
