# Supplementary material for: Development of Hypertolerant Strain of Yarrowia lipolytica Accumulating Succinic Acid Using High Levels of Acetate
Source: ACS Sustain Chem Eng. 2022 Aug 9;10(33):10858–69. doi: 10.1021/acssuschemeng.2c02408 (PMC9400109; doi:10.1021/acssuschemeng.2c02408)
Supplement: Supplementary file 1 — sc2c02408_si_001.pdf [file sc2c02408_si_001.pdf]

Development of hyper-tolerant strain of *Yarrowia lipolytica* accumulating succinic acid using high levels of acetate

Vivek Narisetty[a], Ashish A Prabhu[a], Rajesh Reddy Bommareddy[b], Rylan Cox[c], Deepti Agrawal[d], Ashish Misra[e], M Ali Haider[f], Amit Bhatnagar[g], Ashok Pandey[h,I,j], and Vinod Kumar[a,f*]

[a] School of Water, Energy and Environment, Cranfield University, Cranfield MK43 0AL, United Kingdom

[b] Department of Applied Sciences, Northumbria University, Newcastle upon Tyne, NE1 8ST, United Kingdom

[c] School of Aerospace, Transport and Manufacturing, Cranfield University, Cranfield MK43 0AL, United Kingdom

[d] Biochemistry and Biotechnology Area, Material Resource Efficiency Division, CSIR- Indian Institute of Petroleum, Mohkampur, Dehradun 248005, India

[e] Department of Biochemical Engineering& Biotechnology, Indian Institute of Technology Delhi, New Delhi 110016, India

[f] Department of Chemical Engineering, Indian Institute of Technology Delhi, New Delhi 110016, India

[g] Department of Separation Science, LUT School of Engineering Science, LUT University, Sammonkatu 12, FI-50130, Mikkeli, Finland

[h] Centre for Innovation and Translational Research, CSIR-Indian Institute of Toxicology Research, Lucknow-226 001, India

[i] Centre for Energy and Environmental Sustainability, Lucknow-226 029, India

[j] Sustainability Cluster, School of Engineering, University of Petroleum and Energy Studies, Dehradun-248 007, India

*Corresponding author

Phone: +44(0)1234754786

E-mail: Vinod.Kumar@cranfield.ac.uk

Number of Pages: S10
Number of Supplementary Figures: 05 (S1 – S5)
Number of Supplementary Tables: 02 (S1 – S2)

Figure S1: The Plasmid map of JMP62 LeuTEF containing *acs* gene for transformation of *Y. lipolytica*.

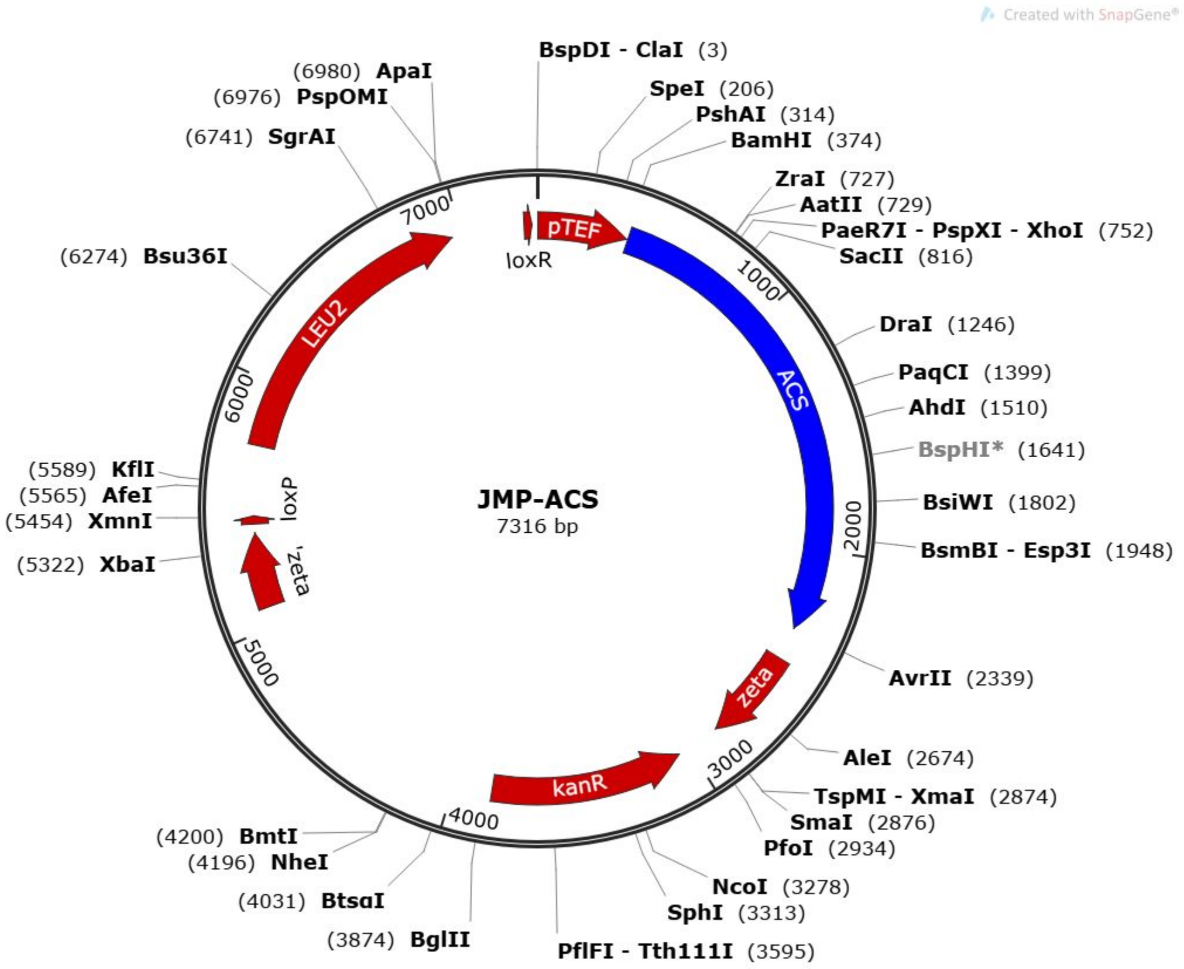

Figure S2: SA production performance by *Y. lipolytica* on acetate. Blue circles represent the elementary modes that lead to biomass formation or SA production. Values represent the yields on acetate.

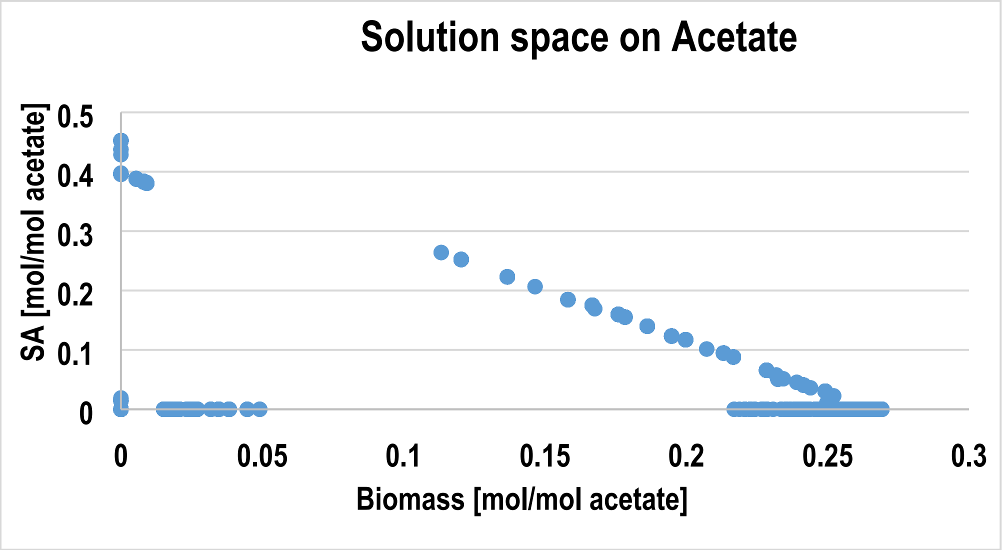

Figure S3: SA production performance by *Y. lipolytica* on glucose. Blue circles represent the elementary modes that lead to biomass formation or SA production. Values represent the yields on glucose.

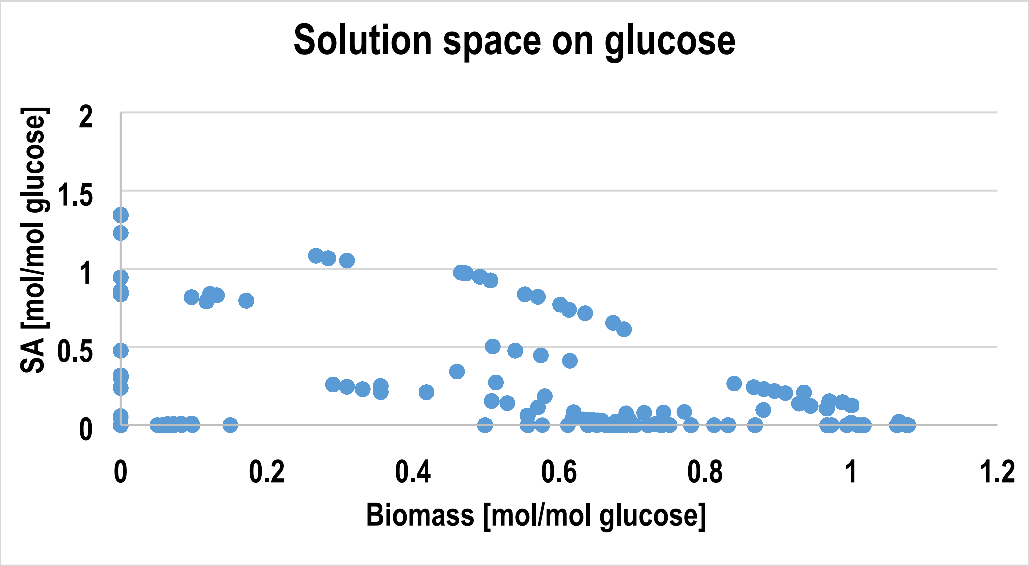

Figure S4: SA production performance by *Y. lipolytica* on glycerol. Blue circles represent the elementary modes that lead to biomass formation or SA production. Values represent the yields on glycerol.

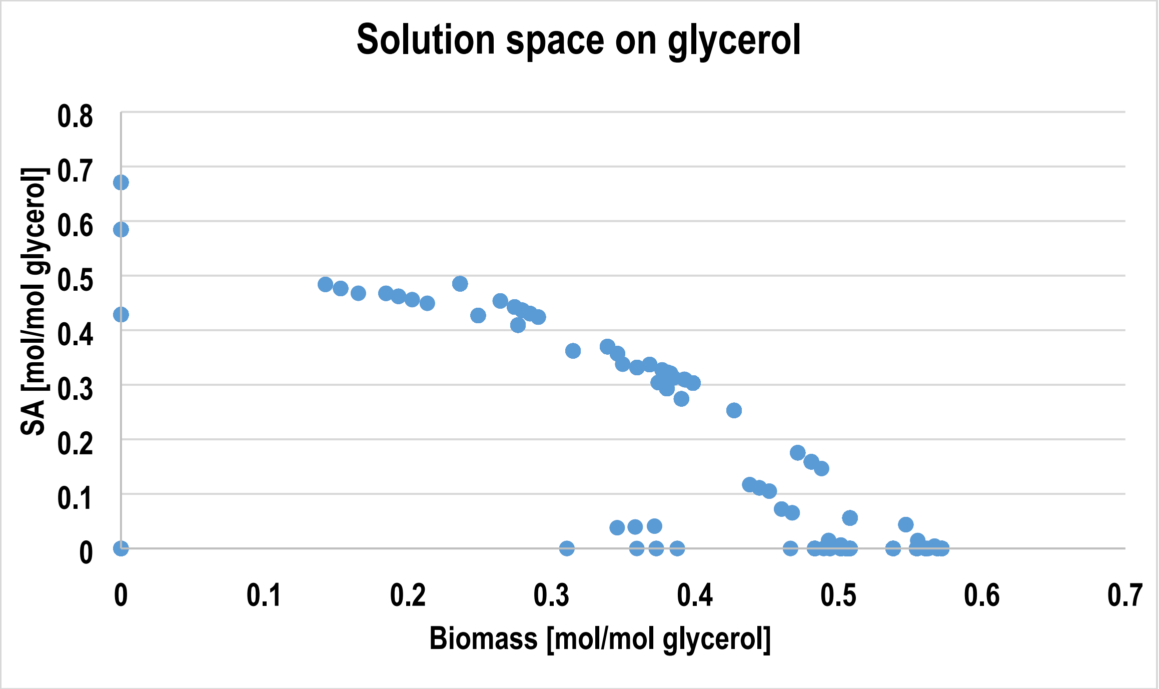

Figure S5: A HPLC Chromatogram representing the consumption of acetate and bioproduction of SA

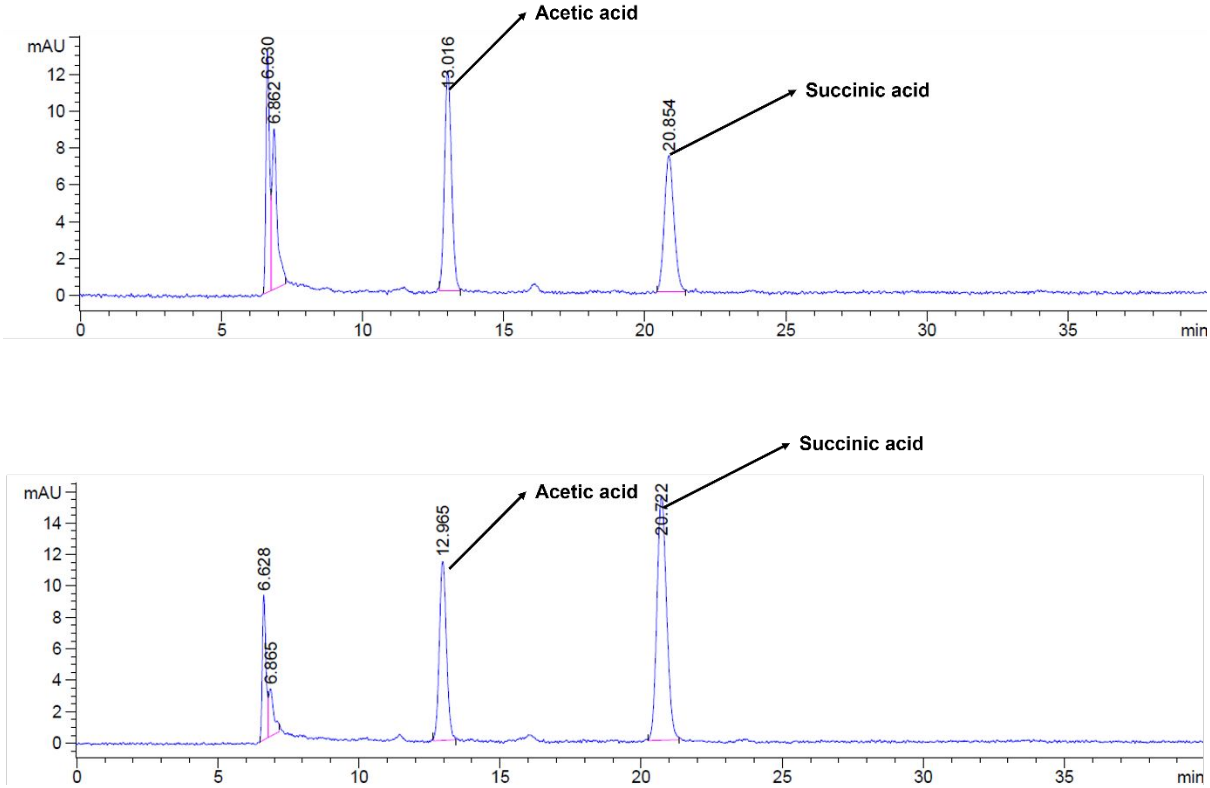

Table S1: Metabolic network of *Y. lipolytica* growing on acetate, glucose, and glycerol; The metabolic reactions are obtained from the previous studies.[1,2]

Enzyme description	ORF	Reaction
**Influx**		
Hexose transporter (HXT)		→ Glucose [e]
Transport		→ Glycerol [e]
Transport		→ Acetate [e]
Transport		→ NH_3 [c]
Transport		→ SO_4 [c]
Transport		→ O_2
**Efflux**		
TAG export		TAG [c] →
Succinate export		Succinate [c] →
Growth (mmol/gDCW*h)		(0.1) Biomass [c] →
ATP for maintenance		ATP maintenance [c] →
Carbon dioxide excretion		CO_2 →
**Glycolysis**		
Hexokinase (HXK1)	YALI0B22308p;YALI0E15488p	GLC [c] + ATP → G6P [c] + ADP
Glucose-6-phosphate isomerase (PGI1)	YALI0F07711p	G6P [c] ↔ F6P [c]
6-phosphofructokinase (PFK1)	YALI0D16357p	F6P [c] + ATP → FBP [c] + ADP
Fructose-bisphosphate aldolase (FBA1)	YALI0E26004p	FBP [c] ↔ GAP [c] + DHAP [c]
Triosephosphate isomerase (TPI1)	YALI0F05214p	DHAP [c] ↔ GAP [c]
Glyceraldehyde 3-phosphate dehydrogenase (TDH)	YALI0C06369p	GAP [c] + NAD [c] ↔ 13-PG [c] + NADH [c]
Glycerol-3-phosphate dehydrogenase (GPD1)	YALI0B02948p	DHAP [c] + NADH [c] ↔ NAD [c] + GLYC3P [c]
Phosphoglycerate kinase (PGK1)	YALI0D12400p	ADP + 13-PG [c] ↔ ATP + 3-PG [c]
Phosphoglycerate mutase (GPM1)	YALI0B02728p	3-PG [c] ↔ 2-PG [c]
Enolase (ENO1)	YALI0F16819p	2-PG [c] ↔ PEP [c]
Pyruvate kinase (PYK1)	YALI0F09185p	PEP [c] + ADP → PYR [c] + ATP
**Pentose phosphate pathway**		
Glucose -6-phosphate 1-dehydrogenase (ZWF1)	YALI0E22649p	G6P [c] + NADP [c] → 6-P-Gluconate [c] + NADPH [c]
6-phosphogluconate dehydrogenase (GND)	YALI0B15598p	6-P-Gluconate [c] + NADP [c] → RIB-5P [c] + CO2 + NADPH [c]
Ribose 5-phosphate isomerase A (RKI1)	YALI0B06941p	Ribulose-5-P [c] ↔ Ribose-5-P [c]
Ribulose-phosphate 3-epimerase (RPE1)	YALI0C11880p	Ribulose-5-P [c] ↔ Xylulose-5-P [c]
Transketolase (TKL1,2)	YALI0D02277p	Ribulose-5-P [c] + Xylulose-5-P [c] ↔

		GAP [c] + S7P [c]
Transaldolase (NOM1)	YALI0F15587p	GAP [c] + S7P [c] ↔ E4P [c] + F6P [c]
Transketolase (TKL1,2)	YALI0E06479p	E4P [c] + Xylulose-5P [c] ↔ GAP [c] + F6P [c]
**TCA cycle**		
Pyruvate dehydrogenase complex (PDB, LAT1)	YALI0E27005p	PYR [m] + NAD [m] → AcCoA [m] + NADH [m] + CO_2
Citrate synthase (CIT1)	YALI0E00638p	AcCoA [m] + OAA [m] → CIT [m]
Aconitate hydratase 1(ACO1)	YALI0D09361p	CIT [m] ↔ ICI [m]
NAD-Isocitrate dehydrogenase (IDH1)	YALI0D06303p	ICI [m] + NAD [m] → AKG [m] + CO_2 + NADH [m]
NADP-Isocitrate dehydrogenase (IDP1)	YALI0F04095p	ICI [m] + NADP [m] → AKG [m] + CO_2 + NADPH [m]
α-ketoglutarate dehydrogenase (KGD1)	YALI0E33517p; YALI0E16929p	AKG [m] + NAD [m] → SUCC-CoA [m] + NADH [m] + CO_2
Succinyl-CoA ligase (LSC1,2)	YALI0E24013p; YALI0D04741p	SUCC-CoA [m] + ADP ↔ SUCC [m] + ATP
Succinate dehydrogenase complex (SDH1-4)	YALI0A14784p	SA [m] + FAD [m] ↔ FUM [m] + FADH [m]
Fumarate hydratase (FUM1)	YALI0C06776p	FUM [m] ↔ MAL [m]
Malate dehydrogenase (MDH1)	YALI0D16753p	MAL [m] + NAD [m] ↔ OAA [m] + NADH [m]
NADP-Malic enzyme (MAE1)	YALI0E18634p	MAL [m] + NADP → PYR [m] + NADPH [m] + CO_2
**Acetyl-CoA formation in cytosol**		
Acetyl-CoA synthetase (ACS)	YALI0F05962g	ATP + Acetate [c] → AMP + AcCoA [c]
ATP:citrate lyase (ACL1)	YALI0D24431p	CIT [c] + ATP → AcCoA [c] + ADP + OAA [c]
**Anaplerotic and gluconeogenic reactions**		
Pyruvate carboxylase (PYC1)	YALI0C24101p	PYR [c] + ATP + CO_2 → OAA [c] + ADP
Phosphoenolpyruvate carboxykinase (PCK1)	YALI0C16995p	OAA [c] + ATP → PEP [c] + ADP + CO_2
Fructose-1,6-bisphosphatase (FBP1)	YALI0A15972p	FBP [c] → F6P [c]
**Glyoxylate cycle**		
Isocitrate lyase (ICL1,2)	YALI0C16885p; YALI0F31999p	ICI [c] → Glyoxy [c] + SA [c]
Malate synthase (MLS1,2)	YALI0D19140p; YALI0E15708p	Glyoxy [c] + AcCoA [c] → MAL [c]
**Glycerol uptake**		
Glycerol kinase (GUT1)	YALI0F00484p	Glycerol [c] + ATP → GLYC3P [c] + ADP
Mitochondrial Glycerol-3-phosphate	YALI0B02948p	GLYC3P [c] + FAD [m] → HAP [c] + FADH[m]

dehydrogenase (GUT2)		
**TAG synthesis**		
Fatty acid (C16) net reaction	--	(8) AcCoA [c] + (7) ATP + (14) NADPH [c] → C16-PAL [c] + (7) ADP + (14) NADP
TAG (C51) net reaction	--	GLYC3P [c] + (3) C16-PA L[c] → TAG [c]
**Oxidative phosphorylation and ATP maintenance**		
NADH dehydrogenase and ATP synthase (NDE1, ATP1,2,16)	YALI0A01419p; YALI0E32164p	(20) NADH [m] + (24) ADP + (10) O_2 → (20) NAD [m] + (24) ATP
Succinate dehydrogenase complex (SDH1-4)	YALI0E29667p; YALI0A14784p	(20) FADH [m] + (24) ADP + (10) O_2 → (20) FAD [m] + (24) ATP
ATP maintenance	--	ATP → ADP + ATP maintenance [c]
Adenylate kinase (ADK1,2)	YALI0B00704p	AMP + ATP → (2) ADP
**Cytosolic reactions and membrane transport reactions**		
Malate dehydrogenase	YALI0E14190p	NADH [c] + OAA [c] ↔ NAD [c] + MAL [c]
Fumarate hydratase	YALI0C06776p	MAL [c] ↔ FUM [c]
Fumarate reductase	YALI0F11957p	NADH [c] + FUM [c] ↔ NAD [c] + SA [c]
Citrate synthase	YALI0E02684p	AcCoA [c] + OAA [c] → CIT [c]
Isocitrate dehydrogenase	YALI0E05137p	ICI [c] + NADP → AKG [c] + NADPH [c] + CO_2
Aconitate hydratase	YALI0D09361p	CIT [c] ↔ ICI [c]
Mitochondrial pyruvate carrier	YALI0F00264p	PYR [c] ↔ PYR [m]
Mitochondrial OAA transporter	YALI0B03344p	OAA [c] ↔ OAA [m]
Mitochondrial dicarboxylate transporter	YALI0D02629p	MAL [c] ↔ MAL [m]
Carnitine-O-acetyltransferase (active only on non-sugar substrates)	YALI0A20988p	AcCoA [c] → AcCoA [m]
Mitochondrial citrate transporter	YALI0F20966p	CIT [c] + MAL [m] ↔ CIT [m] + MA L[c]
Mitochondrial citrate transporter	YALI0F26323p	ICI [m] + CIT [c] ↔ ICI [c] + CIT [m]
Mitochondrial succinate-malate transporter	YALI0F15609p	SA [m] + MAL [c] → MAL [c] + SA [c]
Mitochondrial dicarboxylate transporter	YALI0F00418p	SA [c] → SA [m]
Mitochondrial fumarate-malate transporter	YALI0D06798p	FUM [c] + MAL [m] → MAL [c] + FUM [m]
NAD-NADH shuttle	--	NADH [c] + NAD [m] → NADH [m] + NAD [c]

**Sulphate assimilation**		
Sulphate uptake	YALI0F23551p	SO_4 [c] + (4) NADPH[c] + (3) ATP → H_2S [c] + (4) NADP + (3) ADP
**Ammonia assimilation**		
Glutamate synthetase (GLT1)	YALI0B19998	AKG [c] + GLUM [c] + NADH [c] → (2) GLUT [c] + NAD [c]
Glutamine synthetase (GLN1)	YALI0F00506p	ATP + NH_3 [c] + GLUT [c] → GLUM [c] + ADP
**Biomass formation**		
Biomass formation	--	(10) OAA [c] + (6) 3-PG [c] + (3.2) Ribose-5P [c] + (254) ATP + (90) NADPH [c] + (22) NADPH [m] + (16) NAD [c] + (6) NAD [m] + (24) AcCoA [c] + (3) AcCoA [m] + (18) PYR [c] + (11) AKG [m] + (3.2) E4P [c] + (6.2) PEP [c] + (1.53) H2S [c] + (4.5) F6P [c] + (25) G6P [c] + GLYC3P [c] → biomass [c] + (22) NADP + (90) NADP + (16) NADH [c] + (6) NADH [m] + (254) ADP

Elementary mode and flux analysis: Given a metabolic network with q (70 reactions) and p (61 metabolites), a stoichiometric matrix (q X p) N can be obtained. The set of steady state flux vectors r form the convex fluxes, which can be writes as:

F = [$r \in R^q$ Nr = 0; $r_i \geq 0$, ∀I ∈ Irrev]

Where a set of Irrev of irreversible reactions. As described in Schuster et al., 2000 EMs correspond to minimal functional units (pathways or cycles) of a metabolic network and are useful to study various functional network properties.[3] For flux estimation, the approach utilizes constraints imposed by the biochemistry, a pseudo-steady-state approximation for intracellular metabolites, and the measured accumulation rates of extracellular metabolites to generate flux distribution maps during the course of the fermentation.[4] The set of equations after imposing constraints using measured rates (table 2), a matrix notation of Ax(t) = r(t), where A is a stoichiometric matrix as described above, x(t) is a reaction rate flux vector and r(t) is a metabolite accumulation vector. As the system is underdetermined, matrix notation for solving underdetermined systems was implemented.[5] Based on the above model, a 70 X 61 stoichiometric matrix is obtained where EFMs and flux distributions were elucidated using the software package CellNetAnalyser in Matlab.

Table S2: Molar values of TCA cycle intermediates extracellular flux in *Y. lipolytica* grown on acetate as the carbon source.

**Extracellular fluxes (mol/mol*100)**	
Acetate	100
Biomass	0.90
Acetyl-CoA	9.53
Glycerol-3-phosphate	0.35
NADPH	15.8
Succinate	14.0

References

(1) Sabra, W.; Bommareddy, R. R.; Maheshwari, G.; Papanikolaou, S.; Zeng, A. P. Substrates and Oxygen Dependent Citric Acid Production by Yarrowia Lipolytica: Insights through Transcriptome and Fluxome Analyses. *Microbial Cell Factories* **2017**, *16* (1), 1–14. https://doi.org/10.1186/s12934-017-0690-0.

(2) Pan, P.; Hua, Q. Reconstruction and In Silico Analysis of Metabolic Network for an Oleaginous Yeast, Yarrowia Lipolytica. *PLoS ONE* **2012**, *7* (12), e51535. https://doi.org/10.1371/journal.pone.0051535.

(3) Schuster, S.; Fell, D. A.; Dandekar, T. A General Definition of Metabolic Pathways Useful for Systematic Organization and Analysis of Complex Metabolic Networks. *Nature Biotechnology* **2000**, *18* (3), 326–332. https://doi.org/10.1038/73786.

(4) Vallino, J. J.; Stephanopoulos, G. Metabolic Flux Distributions InCorynebacterium Glutamicum during Growth and Lysine Overproduction. *Biotechnology and Bioengineering* **2000**, *67* (6), 872–885. https://doi.org/10.1002/(SICI)1097-0290(20000320)67:6<872::AID-BIT21>3.0.CO;2-X.

(5) Gregory N. Stephanopoulos, Aristos A. Aristidou, J. N. *Metabolic Engineering: Principles and Methodologies*, 1st ed.; Gregory N. Stephanopoulos, Aristos A. Aristidou, J. N., Ed.; Academic Press, 1998.
